# Reliability and Validity of a New Toileting Assessment Form for Patients with Hemiparetic Stroke

**DOI:** 10.1002/pmrj.12407

**Published:** 2020-06-25

**Authors:** Shin Kitamura, Yohei Otaka, Yudai Murayama, Kazuki Ushizawa, Yuya Narita, Naho Nakatsukasa, Kunitsugu Kondo, Sachiko Sakata

**Affiliations:** ^1^ Department of Rehabilitation Medicine Tokyo Bay Rehabilitation Hospital Chiba Japan; ^2^ Department of Rehabilitation Medicine I, School of Medicine Fujita Health University Aichi Japan

## Abstract

**Background:**

Acquiring toileting independence is an important target of stroke rehabilitation. In planning an intervention for acquiring toileting independence, developing an assessment for individual subtasks that comprise toileting would assist in identifying specific tasks that prevent independence in patients and would facilitate interventions to improve toileting independence.

**Objective:**

To examine the reliability and validity of a newly developed toileting assessment form, the Toileting Tasks Assessment Form (TTAF), for assessing toileting subtasks in hemiparetic stroke.

**Design:**

Validation and test‐retest study.

**Setting:**

Subacute rehabilitation wards in Japan.

**Participants:**

Eighty‐two therapists verified the form's content validity; 30 stroke patients who were using a wheelchair participated in the validation and test‐retest study.

**Interventions:**

Not applicable.

**Main Outcome Measures:**

The content validity of the assessment form was initially assessed based on a questionnaire. Subsequently, four occupational therapists used the form to evaluate video‐recorded toileting performances simulated by participants with hemiparetic stroke. Two assessors evaluated each video‐recorded performance once and repeated the evaluation of the same performance at 2 weeks later. The interrater reliability, intrarater reliability, internal consistency, and concurrent validity of the form were examined.

**Results:**

Fleiss’ κ coefficient for interrater reliability for each form item was 0.61 or more. Cohen's κ coefficient for intrarater reliability for each item was 0.60 or more. Cronbach's coefficient alpha ranged from 0.94 to 0.95. Spearman's rank correlation coefficients for the mean score on the form and the Functional Independence Measure (FIM) score for “toileting” ranged from 0.88 to 0.93 (*P* < .001). Spearman's rank correlation coefficients for the mean score on the form and the FIM score for “toilet transfer” ranged from 0.91 to 0.93 (*P* < .001).

**Conclusions:**

The TTAF demonstrated good reliability and validity. Further multicenter studies involving patients at different stroke phases are required to verify the reliability and validity of TTAF and confirm the generalizability of these findings.

## Introduction

Toileting is a frequent activity of daily living (ADL). Therefore, toileting independence is considered an important determinant for stroke patients who desire to be discharged to their own homes.^1^, ^2^, ^3^ Assistance for toileting is an important and stressful problem that is reported by caregivers of stroke survivors.^4^ Thus, toileting independence is an important target for stroke rehabilitation, and an appropriate evaluation of toileting independence is required for planning rehabilitation.

Instruments of ADL, such as the Barthel Index^5^ and the Functional Independence Measure (FIM),^6^ are frequently used to assess toileting. These established instruments are useful for judging independence and the assistance required for the entire toileting task. However, generic ADL instruments do not provide a breakdown of the individual subtasks that comprise toileting. It has been suggested that an assessment of the individual components of each daily activity is effective for determining rehabilitation goals and treatment planning.^7^ This type of assessment tool has been developed for dressing. A detailed assessment of component tasks of upper‐body dressing can include the most difficult components of dressing and assess the motor skills required for dressing independence.^8^ Furthermore, for toileting independence, both direct tasks, such as pulling the lower garments up or down and transferring to the toilet seat, and associated tasks, such as operating a wheelchair and opening and closing the bathroom door, are required.^9^ In planning an intervention for achieving toileting independence in patients, clinicians should assess a series of subtasks, including the aforementioned associated tasks.

To the best of our knowledge, there is no assessment tool for the series of subtasks that comprise toileting, including not only direct tasks such as pulling the lower garments up or down but also transferring, operating the wheelchair, and going to or from the bathroom. An assessment for a series of individual tasks that comprise toileting management would aid us in identifying specific tasks that prevent independence in patients with hemiparetic stroke and would facilitate interventions to improve toileting independence. This study aimed to verify the reliability and validity of a toileting assessment form, the Toileting Tasks Assessment Form (TTAF), which we had developed and used in our hospital to evaluate independence in various subtasks that comprise toileting among patients with hemiparetic stroke who are using a wheelchair.

## Methods

### *Study Setting and Outline*

The study setting was a 160‐bed subacute rehabilitation hospital in Japan. Patients are eligible for admission to the Kaifukuki Rehabilitation Ward, which is the main system for providing subacute intensive rehabilitation covered by Japan's medical insurance system, within 2 months of stroke onset and can stay up to 6 months after hospitalization.^10^ We developed the TTAF to evaluate the series of subtasks comprising toileting based on expert input. In our hospital, the TTAF had been clinically used for 10 years by occupational therapists to assess the patients’ toileting ability and to share information among medical team members. In the present study, we examined the content validity, interrater reliability, intrarater reliability, and concurrent validity of the TTAF. The protocol for this study was approved by the appropriate ethics committee.

### *Assessment Form*

The TTAF is a tool used to evaluate the toileting activities of patients who experienced a hemiparetic stroke and are using a wheelchair. In the TTAF, toileting subtasks are classified into 24 items. This form was mainly designed to evaluate squat/stand pivot transfer, which is the most common type of transfer for patients with hemiparesis. Nevertheless, the TTAF can be used for other various types of transfer, such as the sliding board transfer, or even for patients who are walking by scoring the items that are not required for transfer as N.

Because this form is to be used for determining specific components of toileting that require intervention in a real‐world environment, all toileting subtasks are sequentially included as they appear in the real situation. Therefore, some items are duplicated. These items include “press the nurse call button,” “turn while standing,” and “maintain a standing position”, which are performed twice during a single toileting activity. These duplicated items are performed in different situations. For example, the situation of the first “press the nurse call button” is that of the need of a staff member to assist transfer to the toilet seat from the wheelchair before urination/defecation; the second one is that of the need of staff member to assist transfer to the wheelchair after excretion. The position of the button on the wall is different for these two situations, and the degrees of urgency are different. This step‐by‐step evaluation of all subtasks during toileting is a distinct characteristic of the form.

### *Content Validity of the TTAF*

Content validity of the TTAF was performed using the Delphi method.^11^ We surveyed the appropriateness of the TTAF using a questionnaire for occupational therapists and physical therapists. Each TTAF item was judged according to whether the assessment could (1) clarify specific steps (or subtasks) that prevent toileting independence, (2) help plan a better rehabilitation program, and (3) be used as a criterion for independence. The responders to the questionnaire were 82 therapists who worked at our hospital. Among them, 38 were occupational therapists (experience, 1‐28 years; median, 3 years) and 44 were physical therapists (experience, 1‐19 years; median, 3 year). The occupational therapists had used the form in the clinical setting. Contrarily, the physical therapists had never used the form before the study. The appropriateness of each TTAF item was judged using a 5‐point Likert scale as follows: 5, very necessary; 4, mostly necessary; 3, neither necessary nor unnecessary; 2, almost unnecessary; and 1, unnecessary. In addition, we asked all responders to indicate the reasons for all items that were scored 3 or less. The form was revised based on the results of the questionnaire. A given item was included if 80% or more of the participants scored it as 4 or 5. All other items were either revised or removed. A second questionnaire‐based survey was conducted to assess the appropriateness of the corrected form. During this survey, participants were allowed to access the overall scores of the first survey for each item. Based on the results of the two surveys, three experts (two occupational therapists and one physiatrist) examined the suitability of each item and finalized the content of the form.

### *Interrater Reliability, Intrarater Reliability, and Concurrent Validity*

Interrater reliability, intrarater reliability, and concurrent validity were examined based on the assessments conducted by four occupational therapists for video‐recorded performances simulated by stroke patients.

### *Participants*

A total of 30 Japanese patients who were admitted to our hospital from June 2016 to June 2018 were enrolled. The inclusion criteria were hemiparetic patients with first‐ever ischemic or hemorrhagic stroke and those who usually used a wheelchair when moving around in the ward, including toileting. For this study, the sample size was determined based on the effect size (*r* = 0.72) reported in a previous study, in which a new ADL instrument for dressing tasks was verified against the FIM.^8^ When the effect size was estimated as *r* = 0.5, a total of 29 participants were required, with power = 0.80 and *α* = 0.05; hence we set the sample size as 30 participants. To include as wide a range of toileting abilities as possible, we recruited the participants by convenience sampling. All participants provided written informed consent prior to their participation in the study. The participants’ background data, including the Stroke Impairment Assessment Set (SIAS) motor items^12^, ^13^ and the FIM,^6^ are presented in Table 1. The SIAS motor items consist of the upper limbs (two items) and lower limbs (three items) on the hemiparetic side, with six grades ranging from 0 (total paralysis) to 5 (normal) for each item. The FIM score evaluated by a nurse within 1 week before or after TTAF evaluation was adopted. As shown in Table 1, the participants had a wide range of paresis severity and ADL.

**Table 1:** Participant characteristics (*n* = 30)

Gender, male/female, *n*	24/6
Age, years, mean (SD)	67.9 (13.8)
Type of stroke, ischemic/intracranial hemorrhage/subarachnoid hemorrhage, *n*	12/17/1
Side of hemiparesis, right/left, *n*	9/21
Days after stroke onset, days, mean (SD)	86.1 (37.7)
Duration of admission to study participation, days, mean (SD)	53.3 (33.8)
Stroke Impairment Assessment Set: Motor Function, median (minimum‐maximum)	
Knee‐mouth	2 (0‐5)
Finger‐function	1 (0‐5)
Hip‐flexion	2 (0‐5)
Knee‐extension	2 (0‐5)
Foot‐pat	2 (0‐5)
Functional Independence Measure, median (minimum‐maximum)	
Motor score	43 (21‐76)
Cognitive score	23 (8‐35)
Total score	65.5 (32‐110)

SD = standard deviation.

### *Assessment for Verifying Reliability and Validity*

The participants were asked to perform a toileting task beginning from their entrance into the bathroom to their exit from the bathroom, except for actual urination and defecation. Assessment was started in front of the closed bathroom door and ended outside the room with the door closed. In the present study, simulation was used for assessment. The participants were instructed to perform a toileting task from opening the door to closing the door in the same manner as actual toileting, except for urination or defecation. We decided not to assess actual urination or defecation in consideration of the participants’ privacy. Using the TTAF, four occupational therapists with 2, 3, 5, and 6 years of clinical experience, respectively, assessed each participant's video‐recorded performance. We adopted a video‐based assessment in view of the following two advantages: First, assessment could be conducted with the same viewpoint of multiple raters; if real‐time assessment is employed, the range that can be seen by multiple raters will be quite different within the very narrow space of the bathroom. Second, assessment through the use of a video could eliminate the influences of the change in participants’ ability during the interval of assessments in the intrarater reliability study.

Each subtask of TTAF was judged as follows: A, independent (the participants can complete the task without intervention from the therapist); B, requires supervision or verbal assistance (the participants are able to complete the task with supervision or verbal assistance from the therapist); C, requires assistance (the therapist needs to physically assist the participants or manipulate the equipment in order for them to complete the task such as braking the wheelchair, transporting the wheelchair to the appropriate position, or pressing the flush button); and N, not applicable (the participants do not need to perform the task; for example, the task of “takes the foot off the footrest and places it on the ground” is applicable only to a wheelchair with a footrest). As for statistical analysis, the following numerical scoring system for the TTAF was employed: A, 3 points; B, 2 points; and C, 1 point. We used the individual score of each item and the mean score of the form to analyze interrater reliability, intrarater reliability, internal consistency, and concurrent validity. The mean score was calculated by dividing the total score by the number of items (excluding the items marked as N [not applicable]).

### *Interrater Reliability*

Fleiss’ κ coefficient was calculated using the assessment scores for each item as marked by the four assessors. The interrater reliability for the mean score given by each assessor was also examined using the intraclass correlation coefficients (ICC [2,1]).

### *Intrarater Reliability*

The assessment was repeated by two assessors with 2 and 3 years of clinical experience, respectively, who had also performed the first assessment following a 2‐week interval. The assessment was performed using the same video as that used for the first assessment. The agreement between each therapist's assessments was evaluated using Cohen's κ coefficient. Furthermore, the intrarater reliability of the mean score was examined using the ICC (1,1). At the mean score, the minimal detectable change with a confidence level of 95% (MDC_95_), the threshold for determining clinical changes beyond measurement error, was also calculated based on the SEM of the intrarater reliability.^14^

### *Internal Consistency*

Cronbach's coefficient alpha for each assessor was calculated based on the item scores of all participants.

### *Concurrent Validity*

Four assessors who scored the TTAF also scored the toileting and toilet transfer of the FIM by watching the same video‐recorded performance. The correlation analyses between the mean scores of the TTAF and the FIM “toileting” scores and between the mean scores of the TTAF and the FIM “toilet transfer” scores were performed for each assessor using the Spearman's rank correlation coefficient.

In addition, to examine whether the TTAF could identify newer aspects of toileting performance compared to the FIM, we compared the score of each TTAF item among participants who had similar FIM “toileting” and FIM “toilet transfer” scores. The median of the TTAF scores and the mode of FIM scores among these four assessors were adopted for this analysis. When calculating this median TTAF score, the “N” was excluded from the calculation if one or two among four assessors judged N for the item, and the “N” was adopted if more than two assessors judged N for the item.

All statistical analyses were performed using the R package (R version 3.3.2). All *P* values <.05 were considered statistically significant. Reliability coefficients were interpreted as follows: slight, 0‐0.20; fair, 0.21‐0.40; moderate, 0.41‐0.60; substantial, 0.61‐0.80; and almost perfect agreement, 0.81‐1.00.^15^

## Results

### *Content Validity*

**Figure 1:**
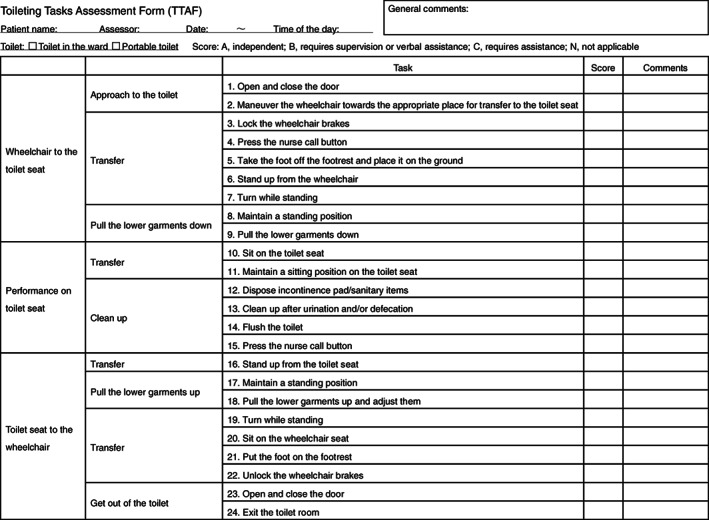
The Toileting Tasks Assessment Form.

The first questionnaire was sent to 82 therapists. Of these 82 therapists, 71 responded (collection rate, 87%). Among the 71 responses, 64 were valid answers (78%; physical therapists, 34; occupational therapists, 29; unknown, 1). All items in the TTAF satisfied the consensus criteria (80% or more of participants scored 4 or 5 for each item). The second questionnaire was also sent to the same 82 therapists. Of these 82 therapists, 67 responded, and all of their responses were valid answers (82%; physical therapists, 33; occupational therapists, 33; unknown, 1). The second questionnaire satisfied the consent criteria for all items, as in the first questionnaire. Therefore, the form was finalized based on the results of the surveys (Figure 1).

### *Interrater Reliability, Intrarater Reliability, and Concurrent Validity*

#### Interrater Reliability

The minimum Fleiss’ κ coefficient value was 0.61, indicating at least substantial interrater reliability for each TTAF item (Table 2). The interrater reliability of the mean score was almost perfect, with an ICC of 0.97 (95% confidence interval [CI], 0.94‐0.98).

**Table 2:** Reliability of each item of the Toileting Tasks Assessment Form (TTAF) (*n* = 30)

Item	Interrater (Fleiss’ κ)	Intrarater (Cohen's κ)	
	Assessors 1‐4	Assessor 1	Assessor 2
1. Open and close the door	0.82	0.94	0.88
2. Maneuver the wheelchair toward the appropriate place for transfer to the toilet seat	0.93	0.87	1.00
3. Lock the wheelchair brakes	0.73	0.94	0.82
4. Press the nurse call button	0.65	1.00	0.66
5. Take the foot off the footrest and place it on the ground	0.80	0.90	0.95
6. Stand up from the wheelchair	0.64	0.70	0.75
7. Turn while standing	0.65	0.95	0.90
8. Maintain a standing position	0.63	0.63	0.74
9. Pull the lower garments down	0.64	0.84	0.90
10. Sit on the toilet seat	0.62	0.95	0.75
11. Maintain a sitting position on the toilet seat	0.66	0.72	0.71
12. Dispose incontinence pad/sanitary items	0.86	0.79	1.00
13. Clean up after urination and/or defecation	0.78	0.80	0.80
14. Flush the toilet	0.82	0.82	1.00
15. Press the nurse call button	1.00	0.66	1.00
16. Stand up from the toilet seat	0.68	0.60	0.75
17. Maintain a standing position	0.61	0.64	0.84
18. Pull the lower garments up and adjust them	0.71	1.00	1.00
19. Turn while standing	0.63	1.00	0.85
20. Sit on the wheelchair seat	0.69	0.67	0.85
21. Put the foot on the footrest	0.91	0.95	0.95
22. Unlock the wheelchair brakes	0.75	0.94	0.89
23. Open and close the door	0.92	0.71	0.93
24. Exit the toilet room	0.74	0.93	0.88

The items are listed in the order of performance.

#### Intrarater Reliability

The minimum Cohen's κ coefficient value for each assessor was 0.60, indicating at least substantial intrarater reliability for each TTAF item (Table 2). The ICC values were 0.99 (95% CI, 0.98‐0.99) and 0.99 (95% CI, 0.99‐0.99) according to the two assessors, respectively, indicating almost perfect intrarater reliability for the mean TTAF score. The SEM was 0.05 (95% CI, 0.04‐0.07) and 0.10 (95% CI, 0.08‐0.13), and MDC_95_ was 0.14 and 0.28 point. These values indicate that if a change of 0.29 point or more is observed when using the TTAF twice for the same participant, the change is considered a “true change” occurring in the participant.

#### Internal Consistency

Cronbach's coefficient alpha values were 0.95, 0.94, 0.94, and 0.95 according to the four assessors, respectively, indicating almost perfect internal consistency.

#### Concurrent Validity

Spearman's rank correlation coefficients for the mean TTAF scores and the FIM “toileting” scores were calculated by the four assessors as 0.91 (*P* < .001), 0.88 (*P* < .001), 0.93 (*P* < .001), and 0.89 (*P* < .001), respectively. Similarly, the coefficients for the mean TTAF scores and the FIM “toilet transfer” scores were calculated by the four assessors as 0.92 (*P* < .001), 0.93 (*P* < .001), 0.93 (*P* < .001), and 0.91 (*P* < .001), respectively.

**Figure 2:**
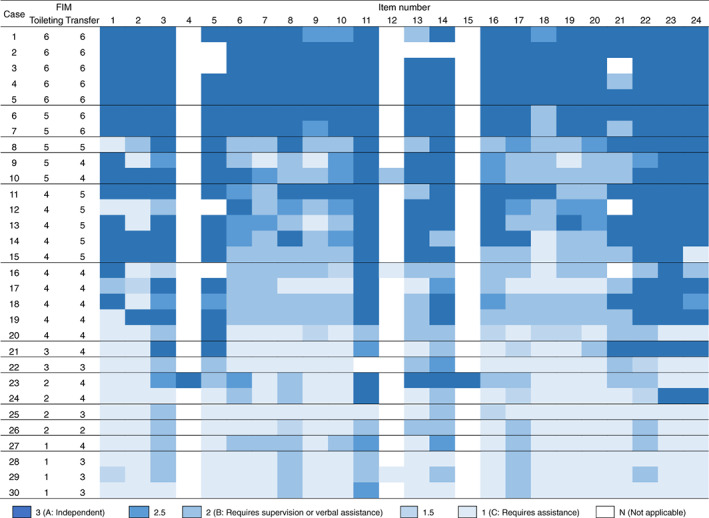
Score of each item of the Toileting Tasks Assessment Form (TTAF) and the scores of the Functional Independence Measure (FIM). The item numbers correspond to the items listed in Table 2. The colors represent the median scores provided for each TTAF item.

The scores of each TTAF item and the FIM scores for “toileting” and “transfer toilet” in individual cases are shown in Figure 2. The results indicated that participants with the same FIM scores had different scores for various TTAF items.

## Discussion

Our new toileting assessment tool, the TTAF, was found to have good content validity, interrater reliability, intrarater reliability, internal consistency, and concurrent validity.

All items of the TTAF satisfied the consensus criteria for content validity according to the questionnaire‐based survey. One possible reason for this favorable response may be that all responders were therapists working in the same hospital where the assessment form was developed. However, among those who responded, only the occupational therapists had previously used the form. Our results suggested that a favorable response was obtained from those who had been using the form in clinical situations and those who were naïve to the form. Therefore, it appeared that our findings on the content validity of the TTAF were acceptable.

Interrater and intrarater reliability values were 0.60 or more for all TTAF items. With respect to interrater reliability, the items for tasks performed in the standing position (stand up from the wheelchair, turn while standing, pull the lower garments down, sit on the toilet seat, and maintain a standing position) were found to have relatively low kappa statistics. Similarly, for intrarater reliability, low kappa statistics were obtained for tasks performed in the standing position (maintain a standing position and stand up from the toilet seat). This may have been related to difficulty in judging between scores of A (independent) and B (requires supervision or verbal assistance) for these tasks. The assessors were required to score participants by watching video recordings of their performance only once. It may be difficult to fully appreciate a participant's stability while performing these tasks, especially those that are performed in the standing position. We presume that this difficulty may be offset during actual clinical situations when the patient's behavior may be evaluated more than once. Furthermore, the therapist may be in a better position to judge the need for assistance in real‐life situations.

During the tests for intrarater reliability, “clean up after urination and/or defecation” was found to have relatively low kappa statistics. In this study, the participants were not video‐recorded during actual urination or defecation. Because toileting was simulated, the participant's performance may have been flawed, contributing to the difficulty in assessing the task accurately. However, this assessment may be reliable in actual clinical situations. We believe that this form may have higher reliability during actual or real clinical situations.

One of the reasons why we developed the assessment form is that there exists no assessment tool that includes not only direct tasks such as pulling the lower garments up or down but also a series of tasks such as entering and exiting the bathroom and operating the wheelchair. Hence, we could not examine the criterion validity against the gold standard. Instead, we verified the concurrent validity against the FIM. Considering that the FIM items “toilet transfer” and “toileting” assess the direct component of toilet transferring and toileting, respectively, the high correlation between these items and the TTAF suggests that the TTAF is valid for assessing the direct parts of tasks related to toilet transfer and toileting.

Participants who had the same FIM “toileting” and “toilet transfer” scores were found to have different scores for various TTAF items, indicating the ability of TTAF to achieve a more detailed evaluation of toileting performance. The FIM score does not identify the specific actions that require assistance. However, the TTAF indicates the degree of independence for individual toileting subtasks. Therefore, in the clinical setting, the TTAF may be more suitable for identifying specific areas of weakness that require intervention. Similar findings were reported by a preliminary correlational study that aimed to develop a new form for assessing subtasks of upper‐body dressing; in this previous study, patients with the same FIM “dressing upper‐body” scores were found to have different scores for the dressing assessment form.^8^ The findings of the previous study and ours suggest that subtask assessment can provide a more detailed evaluation than whole task assessment tools such as the FIM. Furthermore, duplicate items in the TTAF, “turn while standing” (items 7 and 19) and “maintain a standing position” (items 8 and 17), sometimes had different scores even for the same participant (Figure 2). The findings indicate that although the task is the same, it is necessary to sequentially evaluate each task. This is considered one of the strengths of the TTAF compared to traditional assessment tools.

This study had several limitations. We examined simulated and video‐recorded performance instead of conducting in‐person assessment of actual performance, in view of the aforementioned advantages. The results would have been different if in‐person assessment was conducted when the participants performed actual toileting. In fact, the “pushing the nurse call button” items are not required, as toileting was a simulation, the items were evaluated as N in several cases, and reliability could not be sufficiently examined. In addition, the present study was conducted in a single facility, the study sample was limited to subacute stroke patients using a wheelchair, and gender imbalance was rather pronounced (male, 80%). Furthermore, the assessors worked at the same facility where the assessment tool was developed, and the FIM used in this study was not the latest version. Therefore, generalization of the results of this study should be performed with caution. Further multicenter studies in actual clinical settings that involve patients at different stroke phases will confirm the reliability and validity of the TTAF in clinical use.

## Conclusions

This study proved that the TTAF had relatively high reliability and validity. Future studies should explore the usefulness and efficacy of the TTAF for stroke patients in planning and facilitating their rehabilitation.
